# Size-Dependent Sigmoidal Reaction Kinetics for Pyruvic Acid Condensation at the Air–Water Interface in Aqueous Microdroplets

**DOI:** 10.1021/jacs.3c08249

**Published:** 2023-10-03

**Authors:** Meng Li, Christian Boothby, Robert E. Continetti, Vicki H. Grassian

**Affiliations:** Department of Chemistry and Biochemistry, University of California San Diego, La Jolla, California 92093, United States

## Abstract

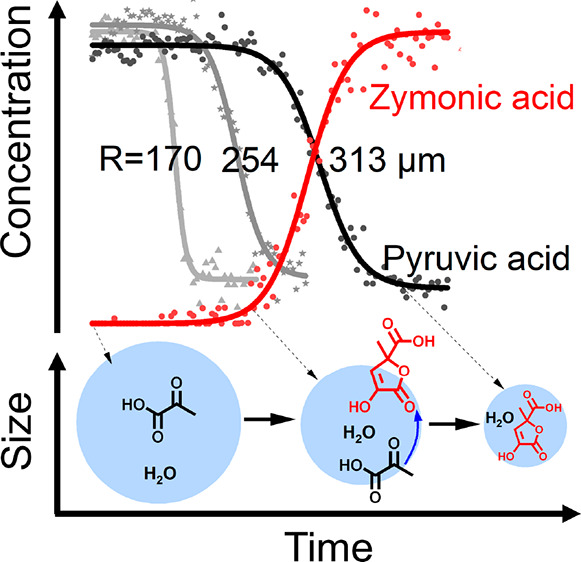

The chemistry of pyruvic acid (PA) under thermal dark conditions is limited in bulk solutions, but in microdroplets it is shown to readily occur. Utilizing in situ micro-Raman spectroscopy as a probe, we investigated the chemistry of PA within aqueous microdroplets in a relative humidity- and temperature-controlled environmental cell. We found that PA undergoes a condensation reaction to yield mostly zymonic acid. Interestingly, the reaction follows a size-dependent sigmoidal kinetic profile, i.e., an induction period followed by reaction and then completion. The induction time is linearly proportional to the surface area (*R*^2^), and the maximum apparent reaction rate is proportional to the surface-to-volume ratio (1/*R*), showing that both the induction and reaction occur at the air–water interface. Furthermore, the droplet size is shown to be dynamic due to changes in droplet composition and re-equilibration with the relative humidity within the environmental cell as the reaction proceeds. Overall, the size-dependent sigmoidal kinetics, shown for the first time in microdroplets, demonstrates the complexity of the reaction mechanism and the importance of the air–water interface in the pyruvic acid condensation reaction.

Aqueous microdroplets have been shown to largely enhance reaction rates and/or initiate reactions that do not occur to any great extent compared to bulk solutions.^1−16^ The high surface-to-volume ratio and the unique environment at the air–water interface have been suggested as the origins of these enhanced rates.^17−21^ Reactions in microdroplets of organic solvents have also been shown to be accelerated compared to bulk solutions.^22,23^

Pyruvic acid (PA) is an abundant α-keto acid in aerosols, fogs, and clouds in the atmosphere, and its conjugate base, pyruvate, is an important intermediate in several metabolic pathways.^24−27^ Its oligomerization can reduce volatility and can contribute to the formation of secondary organic aerosols, impacting air quality and climate.^28,29^ Although PA was reported to spontaneously form zymonic acid (ZA) in bulk solutions in the dark,^30,31^ the reaction is slow, on the order of 20 days.^30^ However, Petters et al.^32^ found a fast volatility change during evaporation of PA in aqueous aerosols, and Kappes et al.^33^ observed the formation of lactic acid and ZA after nebulizing PA solution to small charged PA droplets (60 nm in diameter).

Although there is evidence that shows enhanced reactivity of PA in microdroplets, less is known about the in situ reaction kinetics in microdroplets, and there remains a lack of understanding of the reaction kinetics and mechanisms.^34^ Here we use micro-Raman spectroscopy as an in situ probe of the evolution of the chemical composition and size of PA aqueous microdroplets deposited onto hydrophobic substrates. These substrates are placed in a relative humidity (RH)- and temperature-controlled environmental cell. A schematic of the environmental cell is shown in Figure S1 and the droplet contact angle in Figure S2. Since the evaporation of H_2_O from microdroplets concentrates solutes and may shift the droplet pH, these changes can contribute to enhanced reaction rates compared to bulk solution. To investigate the intrinsic reactivity of PA in microdroplets, the RH is carefully controlled, the microdroplet is in equilibrium with H_2_O vapor, and the initial PA concentration in microdroplets is controlled by the RH. All experiments, except for those investigating the influence of RH, were performed at 95% RH and 22 °C under dark conditions. High-purity nitrogen gas mixtures of wet and dry flows were used to control the RH so that the microdroplet was in equilibrium with H_2_O vapor only.

Figure 1a shows the temporal changes in the Raman spectra during the dark reaction of PA in a single aqueous microdroplet on a hydrophobic substrate. The PA microdroplet is acidic with an initial pH of ca. 1, as determined by a pH calibration curve relating pH to PA concentration obtained from bulk solutions (see Figure S3); this pH is significantly lower than the p*K*_a_ of PA (∼2.4 and 3.6 for keto and diol forms, respectively).^35,36^ Therefore, protonated forms of PA are the major species present. The initial microdroplet radius (*R*_i_) for this experiment was 217 μm. The error in the measured *R*_i_ is on the order of 5 μm. As can be seen in Figure 1a, there is an induction period during which no obvious changes in the spectra are detected (0 to 24 min). After the induction period, there is reaction with changes observed in the Raman spectra indicative of depletion of PA until completion after approximately 69 min, as indicated by the identical Raman spectra afterward. In particular, during the reaction, Raman peaks for PA, ν(C–C) at 785 cm^–1^, ν(C=O) at 1738 cm^–1^, and ν(CH_3_)_sym_ of the PA keto form at 2931 cm^–1^,^37,38^ decrease. Note that some PA peaks, δ(CH_3_) at 1450 cm^–1^, ν(CH_3_)_sym_ of the PA diol form at 2946 cm^–1^, and ν(CH_3_)_asym_ at 3007 cm^–1^,^37,38^ do not change as much as other PA peaks, likely due to their overlap with product peaks. In addition, new peaks grow in, which are indicative of the formation of ZA. For example, the peak at 796 cm^–1^ can be assigned to ν(C–C) adjacent to the carboxylic acid group. The peaks at 1660 and 1686 cm^–1^ originate from different ν(C=C) of different forms of ZA, and peaks at 1774 and 3108 cm^–1^ are due to ν(C=O) and vinylic ν(=C–H) of ZA, respectively.^31,38^ To further characterize the chemical composition of the products, we analyzed microdroplets by NMR spectroscopy and mass spectrometry. Our analysis reveals that the peaks associated with ZA dominate both the ^1^H NMR and mass spectra, as shown in Figures S4 and S5, respectively. These results suggest that ZA is the major product. We also observed the presence of parapyruvic acid (PPA), as indicated by the small peak at *m*/*z* = 175 ([PPA – H]^−^) in the mass spectrum. Moreover, no reactions were observed in a sodium pyruvate microdroplet (*R*_i_ = 212 μm) with a pH of ∼6.8 after 12 h (Figure S6), indicating the role of acidity in the reaction mechanism. Taking these results together, we propose that the PA keto form undergoes acid-catalyzed condensation reactions, namely, aldol addition followed by cyclization, to form ZA (Figure 1b). This agrees with results obtained by Vaida and co-workers on reactions of pyruvic acid.^31,33,39^

**Figure 1:**
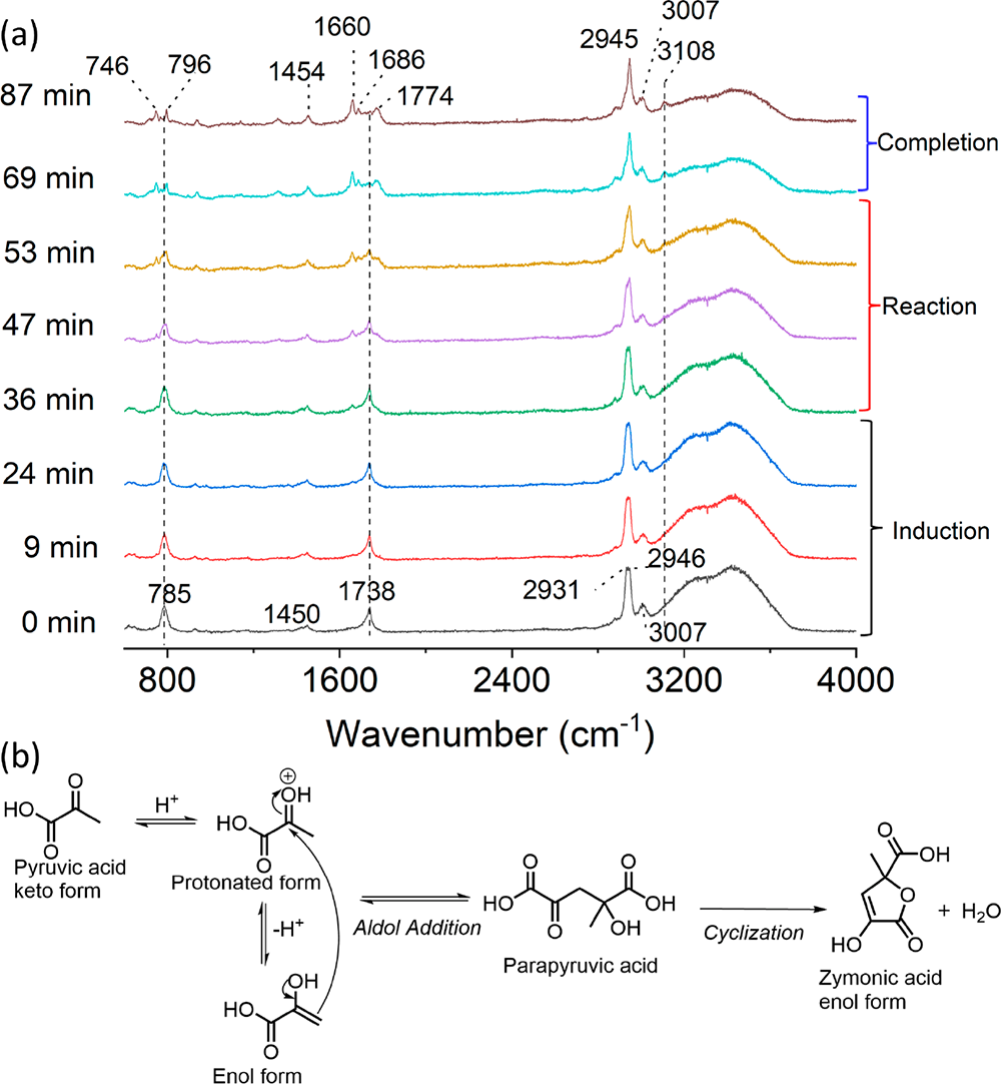
(a) Representative Raman spectrum change during the reaction of pyruvic acid (PA) in an acidic microdroplet with an initial radius (*R*_i_) of 217 μm. The initial PA concentration in the microdroplet is 4.3 mol kg^–1^. (b) Mechanism for the PA condensation reaction.^31,33,39^

To analyze the in situ reaction kinetics of PA in microdroplets, the PA concentration in units of molality (*m*_PA_) is determined from a ratiometric approach (see the discussion in the Supporting Information (SI) and Figure S7). Figure 2a presents the time evolution of *m*_PA_ in a droplet with *R*_i_ = 170 μm. We observed that *m*_PA_ remains relatively constant during the induction period, followed by a rapid decrease and a gradual slowing down until reaction completion, i.e., PA reaction in microdroplets undergoes an induction period followed by a reaction period before completion of the reaction. For product formation, similar kinetics was observed (Figure S8). For bulk solutions, these kinetic profiles of reactant and products represent an autocatalytic reaction.^40−42^

**Figure 2:**
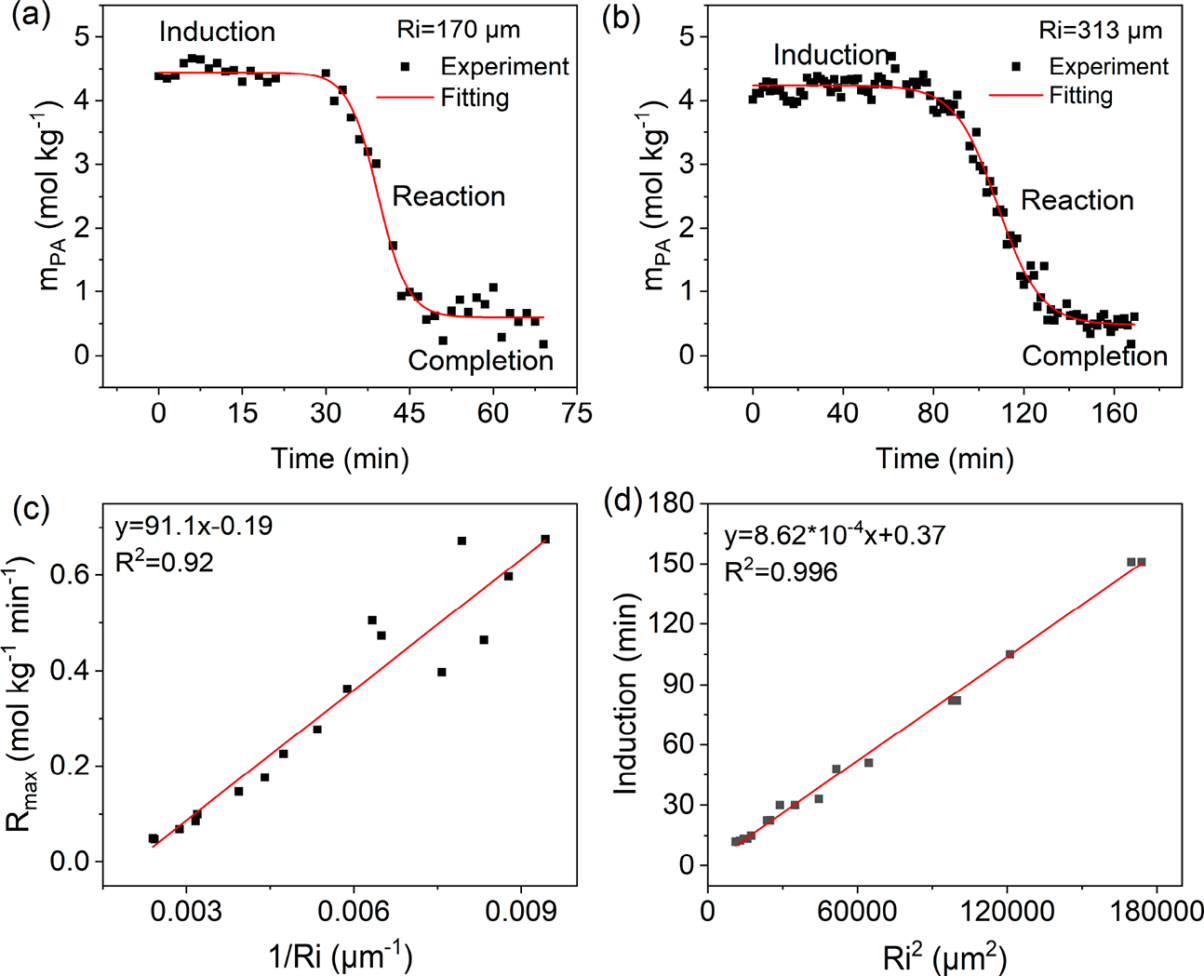
Size-dependent sigmoidal kinetics for PA condensation in microdroplets. (a, b) Time evolution of the PA concentration (*m*_PA_) with initial radii (*R*_i_) of (a) 170 and (b) 313 μm. The squares present experimental data, and the lines are Boltzmann sigmoidal fittings (see the SI for details of the data analysis). (c) Maximum apparent reaction rate (*R*_max_) of PA versus 1/*R*_i_. (d) Induction time versus *R*_i_^2^. Linear fits are shown for (c) and (d).

For a larger droplet with *R*_i_ = 313 μm (Figure 2b), the kinetics is similar; however, the lengths of both the induction period and reaction period are significantly longer compared to those for droplets with *R*_i_ = 170 μm. Both the induction and reaction occur faster in the smaller droplet. The initial *m*_PA_ for both droplets was almost identical, as expected because the RH was the same and the initial *m*_PA_ is controlled by the RH. Therefore, the increased induction and reaction rate in the smaller droplets cannot be attributed to any concentration difference; instead, it arises from the difference in droplet size.

These reaction kinetics can be fit to a Boltzmann sigmoidal equation (see SI). The size-dependent sigmoidal kinetics for individual droplets covering a wide range of radii from 106 to 417 μm were analyzed. The maximum apparent reaction rate, *R*_max,_ as defined in eqs 1–3 in the SI, and induction time (Figure 2c,d) were determined. *R*_max_ of the PA reaction in microdroplets is found to be inversely proportional to *R*_i_. It is accelerated over an order of magnitude, from 0.05 to 0.68 mol kg^–1^ min^–1^, when *R*_i_ decreases from 417 to 106 μm. The induction time shows a linear correlation with *R*_i_^2^. These results, i.e., the surface-to-volume ratio (1/*R*) dependence of the rate and the surface area (*R*^2^) dependence of the induction time, definitively demonstrate that both the induction and reaction of PA condensation reactions occur at the air–water interface. The distinct environment at the air–water interface, such as the solute orientation/structure and changes in the reaction mechanism and reaction barrier, is likely the origin of the reactivity. No significant reactions were observed for bulk solutions with PA concentrations ranging from 4.7 to 141.9 mol kg^–1^ after 7 days (Figure S9). Thus, the reaction is greatly enhanced by 10^3^ to 10^4^ orders of magnitude, depending on the size, relative to the bulk phase.

Furthermore, droplet size is dynamic due to changes in composition and equilibration with RH in the environmental cell. As shown in Figure 3, microdroplets with *R*_i_ from 113 to 415 μm show a decrease in size over time, with smaller droplets exhibiting a higher rate of size reduction. The size evolution profile can be divided into three regions based on the rate of size decrease, i.e., a relatively low rate during the initial period, followed by a higher rate and finally reaching a stable size. These three regions match the induction, reaction, and completion periods observed in the evolution of *m*_PA_ (Figure S10). During the induction period, both PA and H_2_O partition from the droplet with ∼50% PA and H_2_O loss. This is followed by reaction and changes in droplet composition with less hygroscopic products along with a re-equilibration with the RH inside of the environmental cell.^32^ This leads to a decrease in microdroplet water content and size (see SI and Figure S11).

**Figure 3:**
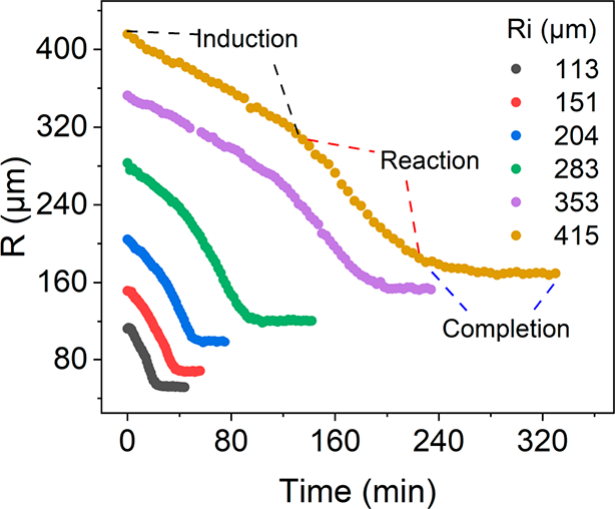
Representative droplet radius (*R*) changes as a function of time during the PA condensation reaction in microdroplets. *R* was determined from the bright-field image of the micro-Raman spectroscopy. The initial PA concentration in the microdroplets was in the range of 4–4.6 mol kg^–1^.

The influence of RH on the sigmoidal reaction kinetics was also investigated here by comparing the time evolution of *m*_PA_ under different RH conditions. In this experiment, droplets with similar *R*_i_ (210 ± 5 μm) were selected. As shown in Figure 4, we directly observed a higher initial *m*_PA_ (during the induction period) at a lower RH. This is expected as the microdroplet equilibrates with the surrounding RH, leading to different *m*_PA_ depending on RH.^43,44^ Additionally, we observed a shorter induction period and a higher apparent reaction rate under lower-RH conditions, where the droplet has less water content. These results indicate that an increase in *m*_PA_ and a decrease in microdroplet water content accelerate the kinetics, both the induction and reaction.

**Figure 4:**
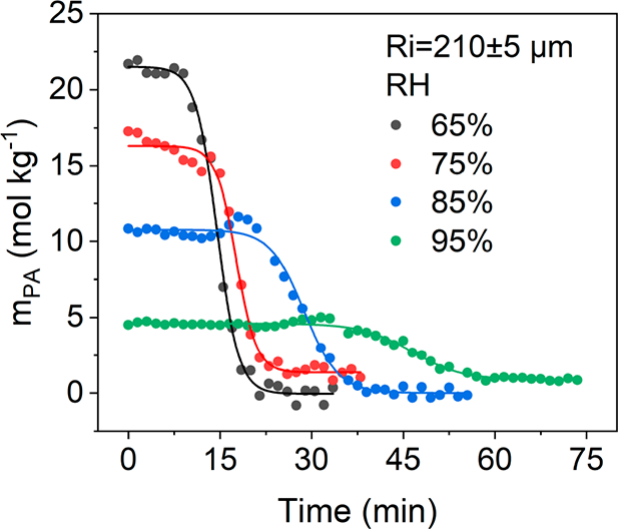
Comparison of the time evolution of *m*_PA_ under 65% (black), 75% (red), 85% (blue), and 95% (green) RH conditions. *R*_i_ = 210 ± 5 μm. The circles present experimental data, and the lines are Boltzmann sigmoidal fittings. Similar behavior of the impact of RH on microdroplets of various sizes is shown in Figure S12.

These results taken together show that the condensation of PA under acidic conditions in microdroplets increases with decreasing microdroplet size and decreasing RH. Importantly, we find that the sigmoidal kinetics is dependent on *R*_i_ in the following manner: (1) the reaction induction time is linearly proportional to surface area (*R*_i_^2^), and (2) *R*_max_ is proportional to the surface-to-volume ratio (1/*R*_i_). These findings conclusively demonstrate that both the induction and reaction occur at the air–water interface. The dependence on RH shows that *R*_max_ increases with lower water content in the microdroplet. Most interesting is the sigmoidal kinetics, which has been observed for the first time, to the best of our knowledge, for microdroplets and, in particular, for the PA condensation reaction. Since the induction period is surface-area-dependent, it is likely that reactive complexes/species form and accumulate at the air–water interface during this period. In bulk solutions with kinetic profiles for reactants and products similar to those observed here, there is an autocatalytic reaction whereby ZA can catalyze the reaction. However, unlike bulk reactions, the loss of H_2_O from the microdroplet as the reaction proceeds, as seen by a decrease in droplet size, can drive the condensation reaction. The dynamic nature of the microdroplet composition, size, and surface reactivity driven in part by the gas-phase H_2_O equilibrium pressure gives rise to complex reaction kinetics as demonstrated here for the first time. Further efforts to disentangle these different effects within aqueous microdroplets (water activity, droplet size, and reaction mechanisms) will be key to understanding aqueous microdroplet reactivity.

## Supporting Information Available

Additional information on materials and methods, including chemicals, generation of microdroplets and micro-Raman spectroscopy analysis, NMR analysis, and mass spectrometric analysis as well as the details and key advantages of the ratiometric approach, Boltzmann sigmoidal fitting, and a discussion about droplet size changes; schematic of the experimental setup (Figure S1); contact angle measurement of droplets on a hydrophobic coated surface (Figure S2); correlation between pH and PA concentration (Figure S3); NMR data (Figure S4); mass spectral data (Figure S5); Raman spectra of sodium pyruvate aqueous microdroplet from 0 to 12 h (Figure S6); calibration curve relating *m*_PA_ to Raman peak area (Figure S7); kinetic data demonstrating sigmoidal kinetics for both reactant depletion and product formation (Figure S8); data for bulk solutions (Figure S9); induction and reaction times as functions of droplet initial radius (Figure S10); ratio of the droplet size to the initial droplet size at the end of induction and at the end of reaction (Figure S11); time evolution of *m*_PA_ under different RH conditions with various droplet sizes (Figure S12) (PDF)
