# Supplementary material for: Size-Dependent Sigmoidal Reaction Kinetics for Pyruvic Acid Condensation at the Air–Water Interface in Aqueous Microdroplets
Source: J Am Chem Soc. 2023 Oct 3;145(41):22317–21. doi: 10.1021/jacs.3c08249 (PMC10591466; doi:10.1021/jacs.3c08249)
Supplement: Supplementary file 1 — ja3c08249_si_001.pdf [file ja3c08249_si_001.pdf]

Supporting information for

Size-Dependent Sigmoidal Reaction Kinetics for Pyruvic Acid Condensation at the Air-Water Interface in Aqueous Microdroplets

Meng Li[a], Christian Boothby[a], Robert E. Continetti[a]* and Vicki H. Grassian[a]*

[a]Department of Chemistry and Biochemistry, University of California San Diego, La Jolla, California 92093, USA.
*Correspondence to: V.H.G. (vhgrassian@ucsd.edu) and REC. (rcontinetti@ucsd.edu)

This file includes:
Supplementary text
Figures S1 to S12
References

Materials and Methods

Chemicals. Pyruvic acid (98%, extra pure, nitrogen flushed) was purchased from Thermo Scientific. Sodium pyruvate (99%) was purchased from Sigma-Aldrich. Methanol (HPLC grade) was purchased from Fisher Scientific and dimethyl sulfoxide-d6 (DMSO-d6, D, 99.9%) was purchased from Cambridge Isotope Laboratories, Inc. Milli-Q water (Millipore Sigma, 18.2 MΩ) was used as the solvent to obtain different concentrations of pyruvic acid (PA) solution.

Generation of microdroplets and micro-Raman spectroscopy analysis. The PA reaction in microdroplets was monitored in situ using micro-Raman spectroscopy (Horiba, LabRamn HR Evolution) coupled to an environmental cell (Linkam, LTS 120), as shown in Figure S1. The spectrometer is fitted with an optical microscope (Olympus BX41), a 10× and a 100× super long working distance objective, and a 532 nm laser. The environmental cell is equipped with a temperature controller (Linkam, T-95 PE), which allows for temperature control. Additionally, the cell has a gas inlet and a gas outlet connected to a hygrometer (Buck, CR-4) to determine the relative humidity (RH).[1] The RH of the environment cell was adjusted by mixing different ratios of dry and humidified N_2 gases. A single aqueous microdroplet was deposited on a hydrophobic coverslip (Figure S2) using a micropipette, and the coverslip was placed inside the environmental cell. The PA solutions used to generate microdroplets had concentrations of 3 mol kg^{-1}, 5 mol kg^{-1}, 6.4 mol kg^{-1}, 10 mol kg^{-1} and 15 mol kg^{-1} for experiments under 95%, 90%, 85%, 75% and 65% RH conditions, respectively. After placing a microdroplet inside the environmental cell, Raman spectra were collected in situ with an acquisition time of 10 s.

NMR analysis. The products from PA microdroplet reaction were analyzed by a 500Hz Jeol ECA NMR Spectrometer. The products were prepared by depositing multiple PA microdroplets (covering a range of radii from 100 to 500 μm) on hydrophobic coverslips. The hydrophobic coverslips with PA microdroplets were placed in the Raman environmental cell under 95% RH at 22 °C. After the reaction is complete (confirmed by Raman spectra), the microdroplets containing products were dissolved in DMSO-d6 for the ^{1}H NMR measurements.

Mass spectrometric analysis. A direct-injection linear ion trap (Thermo Fisher Orbitrap) high-resolution mass spectrometer (HRMS) was used for mass spectrometric analysis of the products obtained from PA reactions in microdroplets. The products were prepared with similar procedures as those in the NMM analysis except for using methanol to dissolve microdroplets from the coverslip after the completion of the reaction. HRMS analysis in negative electrospray ionization (ESI) mode was used. Parameters for the heated ESI source were as follows: Heater temperature, 100 °C; Spray voltage, 3.00 kV; Capillary temperature, 300°C.

Data Analysis

Ratiometric approach for determining PA concentration. To analyze the in-situ reaction kinetics of PA in microdroplets, the peak area ratio of PA (ν(C-C) at 785 cm^{-1}) and H_2O (OH band from 3180 to 3750 cm^{-1}) (A_{PA}/A_{H_2O}) is used to calibrate and determine PA concentration in units of molality (m_{PA}, mol kg^{-1}). The calibration curve is obtained by relating m_{PA} to A_{PA}/A_{H_2O} using bulk solutions with known PA concentrations, and an excellent linear correlation is observed over a wide PA concentration range (0.5 to 15 mol kg^{-1}) (Figure S7). The changes in m_{PA} during PA reactions in microdroplets are determined by converting the A_{PA}/A_{H_2O} of microdroplets obtained from the Raman spectra to m_{PA}, using the calibration curve. This ratiometric approach offers two key advantages. First, it can normalize changes in droplet size and laser power fluctuations. Second, it excludes the contribution of PA evaporation to the decrease in PA concentration, as the A_{PA}/A_{H_2O} reflects the molality of PA, i.e., the molar amount of PA per unit mass of H_2O. Evaporation of PA under a constant RH condition will result in a corresponding evaporation of H_2O, maintaining a constant PA molality. In other words, a decrease in A_{PA}/A_{H_2O} explicitly reflects the decrease of PA concentration caused by reactions.

Boltzmann sigmoidal fitting. The PA reaction kinetics in aqueous microdroplets can be fitted by a Boltzmann sigmoidal equation[2]

$$y = \frac{A_i - A_f}{1 + e^{(x-x_0)/a}} + A_f \quad (1)$$

Where A_i and A_f are the initial and final values of the curve, respectively. In eqn. (1), x_0 is the abscissa of the curve's central inflection point and a is a constant.

The maximum slope (S_{max}) of the curve is at x_0 and determined by

$$S_{max} = (A_f - A_i)/(4a) \quad (2)$$

The maximum reaction rate (R_{max}) for the PA dark reaction is determined by

$$R_{max} = -S_{max} \quad (3)$$

Changes in droplet size. During the induction period, the droplet size slowly decreases while the m_{PA} remains constant. This is because PA is semi-volatile and evaporates from the droplet to the gas phase. Since the microdroplet is in equilibrium with the surrounding RH in the environmental cell, a corresponding amount of H_2O also evaporates from the droplet. Consequently, the microdroplet decreases in size while maintaining a constant m_{PA}. Our analysis shows that all the investigated droplets (113 μm ≤ Ri ≤ 415 μm) exhibit a similar ratio of the size at the end of induction to the initial size, with an average value of 0.79 and a standard deviation of 0.037 (Figure S11). This result indicates that ~50% of PA and H_2O partition from the droplet to the gas phase. During the reaction period, in addition to the evaporation of PA, the formation of less hygroscopic products (mostly ZA) through the reaction of PA, and the re-equilibration with the RH inside the environmental cell, lead to a decrease in water content, further contributing to droplet size reduction. Since ZA has low volatility, it does not evaporate significantly during the completion period, leading to a stable size, in agreement with a recent study.[3] The ratio of the droplet size at the end of reaction to the initial droplet size has an average value of 0.45 with a standard deviation of 0.023 (Figure S11).

Figures

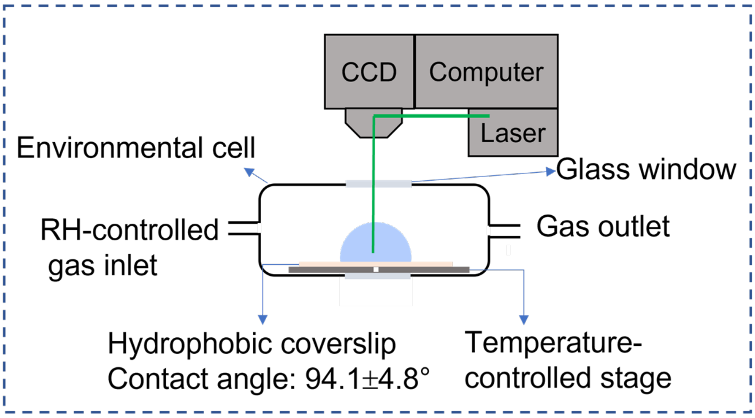

Figure S1. Schematic of the experimental setup.

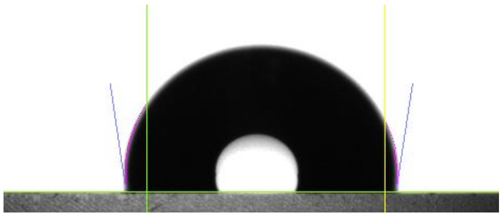

Figure S2. The contact angle of the hydrophobic coverslip. 3 μL of water droplets were used for the contact angle measurements. The contact angle is 94.1±4.8°.

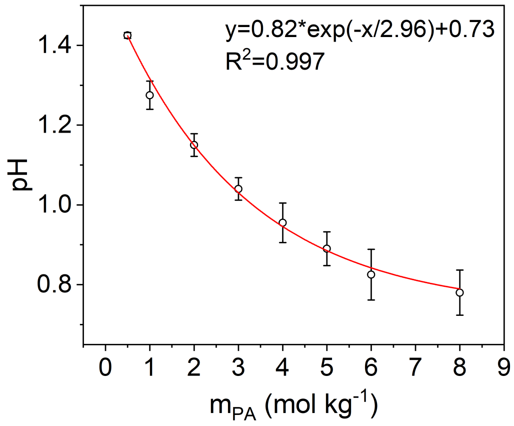

Figure S3. The correlation between the pH and the concentration of PA solution (m_{PA}). The solution pH was measured by a pH meter (Oakton pH 700). The droplet in Figure 1 had an initial m_{PA} of 4.3 mol kg^{-1}, thus, the initial droplet pH is determined to be 0.9 based on the correlation between pH and concentration.

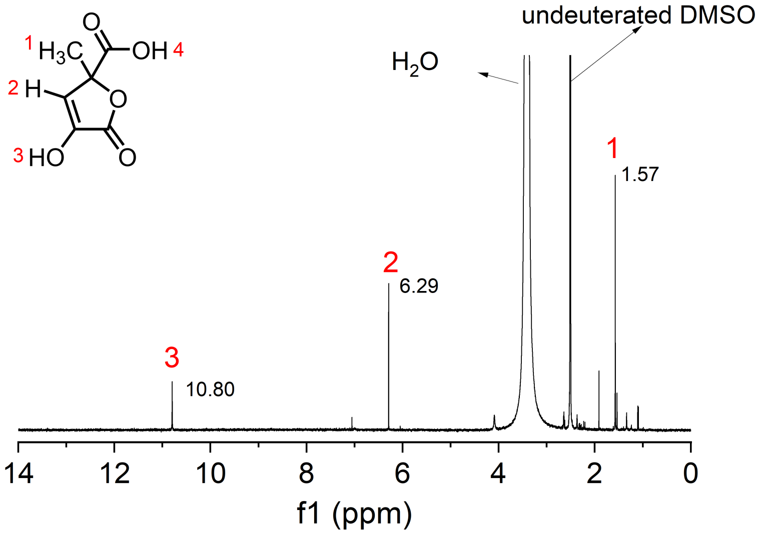

Figure S4. ^{1}H NMR spectrum (500 MHz) of the products from PA microdroplet reaction dissolved in DMSO-d6. The peaks at 1.57, 6.29 and 10.08 ppm are assigned to methyl proton (H1), alkene proton (H2) and the enol proton (H3) in zymonic acid (ZA).[4] The ratios of the peaks H1:H2:H3 are 3.14:1:1.05. The absence of the acid proton (H4) is likely due to the exchange of the acid proton with water in the DMSO.

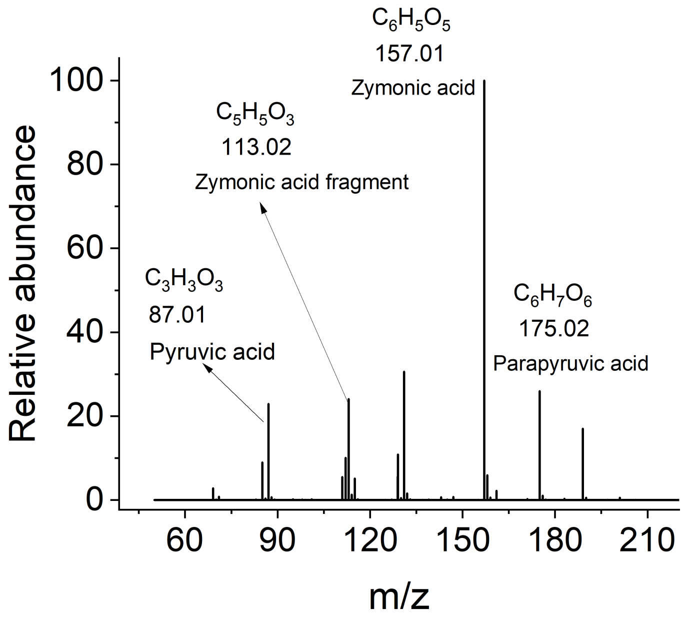

Figure S5. Mass spectrum of the products obtained from PA reaction in microdroplets.

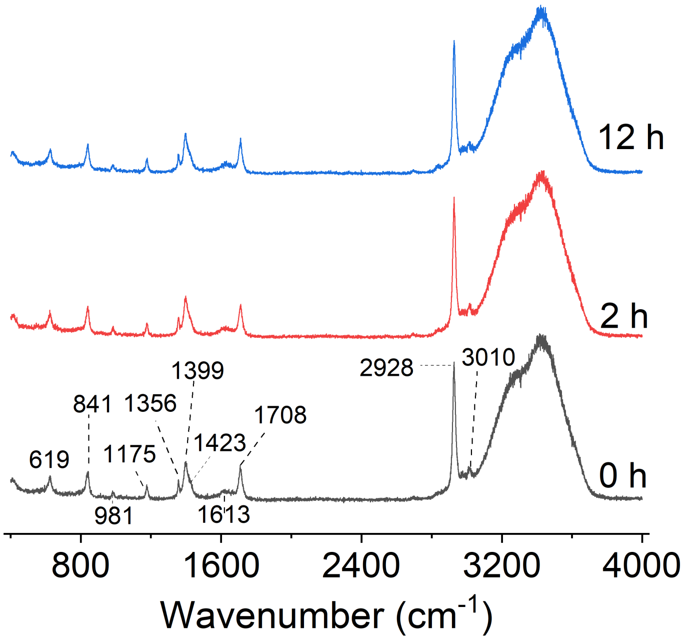

Figure S6. Raman spectra of sodium pyruvate aqueous microdroplet with a radius of 212 μm at 0 h (black), 2 h (red) and 12 h (blue). The peaks of pyruvate (CH_3COCOO^-) from the low wavenumber to high wavenumber are assigned to COO^- wag (619 cm^{-1}), C-C stretch (841 cm^{-1}), C-CH_3 stretch (1175 cm^{-1}), sym. CH_3 bending (1356 cm^{-1}), sym. COO^- stretch (1399 cm^{-1}), asym. CH_3 bending (1423 cm^{-1}), asym. COO^- stretch (1613 cm^{-1}), C=O stretch (1708 cm^{-1}), sym. CH_3 stretch (2928 cm^{-1}), asym. CH_3 stretch (3010 cm^{-1}).[5-7] Note no changes in spectra were detected after 12 hours, indicating that no reaction occurred during that period. The microdroplet was generated from a sodium pyruvate solution with a concentration of 1.8 mol kg^{-1}. The solution had a pH of 6.77±0.02, as measured by the pH meter, and this pH was used to indicate the droplet pH. We are aware that the droplet pH may not be exactly the same as the bulk solution. Nevertheless, the droplet was in equilibrium with a high RH (95%) of in the environmental chamber. The uncertainty of droplet pH caused by using bulk pH as a surrogate in this case should not be significant.

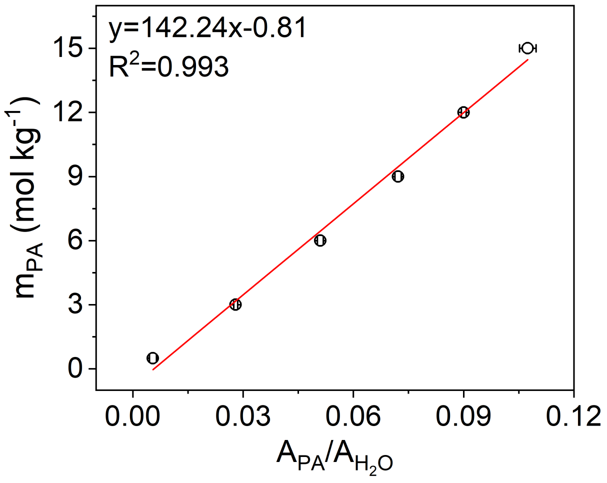

Figure S7. The calibration curve relating m_{PA} to the integrated peak area ratios of PA (ν(C-C), at 785 cm^{-1}) and H_2O (OH band from 3180 to 3750 cm^{-1}) (A_{PA}/A_{H_2O}).

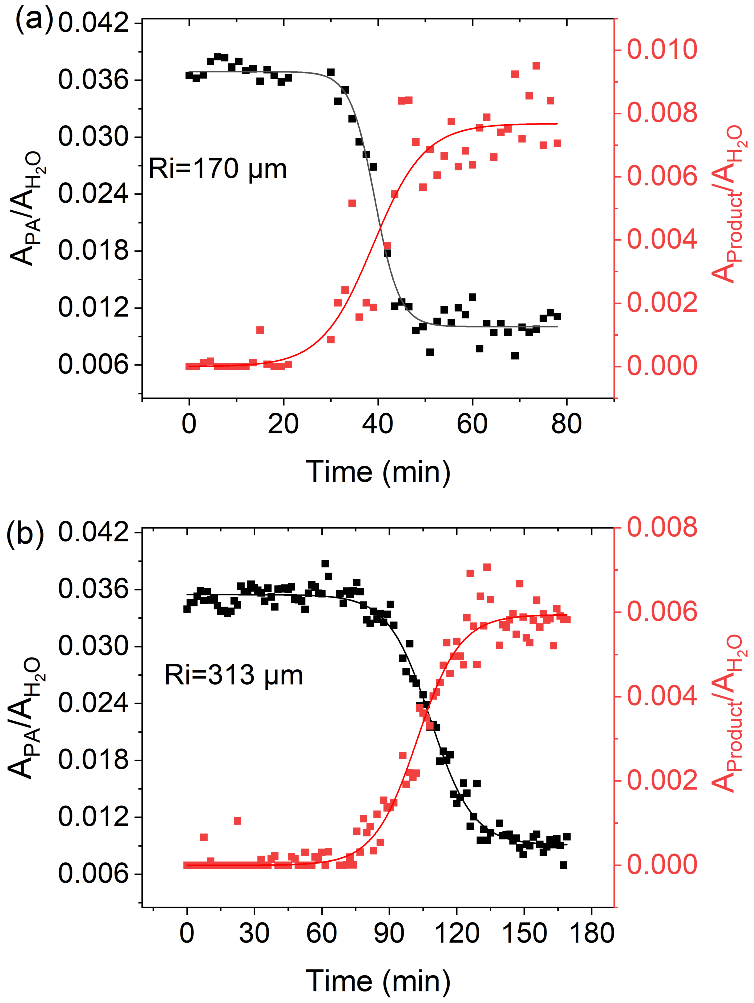

Figure S8. Time evolution of the peak area ratio of PA (at 785 cm^{-1}) and H_2O (from 3180 to 3750 cm^{-1}) (A_{PA}/A_{H_2O}) (black) and the peak area ratio of the product (at 796 cm^{-1}) and H_2O (from 3180 to 3750 cm^{-1}) ($A_{Product}/A_{H_2O}$) (red) in droplets with Ri of (a) 170 μm and (b) 313 μm. Due to the lack of a standard for the product, we used $A_{Product}/A_{H_2O}$ to reflect the concentration of the product. Similarly, we also used A_{PA}/A_{H_2O} to reflect PA concentration, even though we have the PA concentration values (Figure 2a and 2b). The similar shape between the evolution of A_{PA}/A_{H_2O} here and m_{PA} in Figures 2a and 2b, suggests that the peak area ratio is a good choice to reflect the concentration.

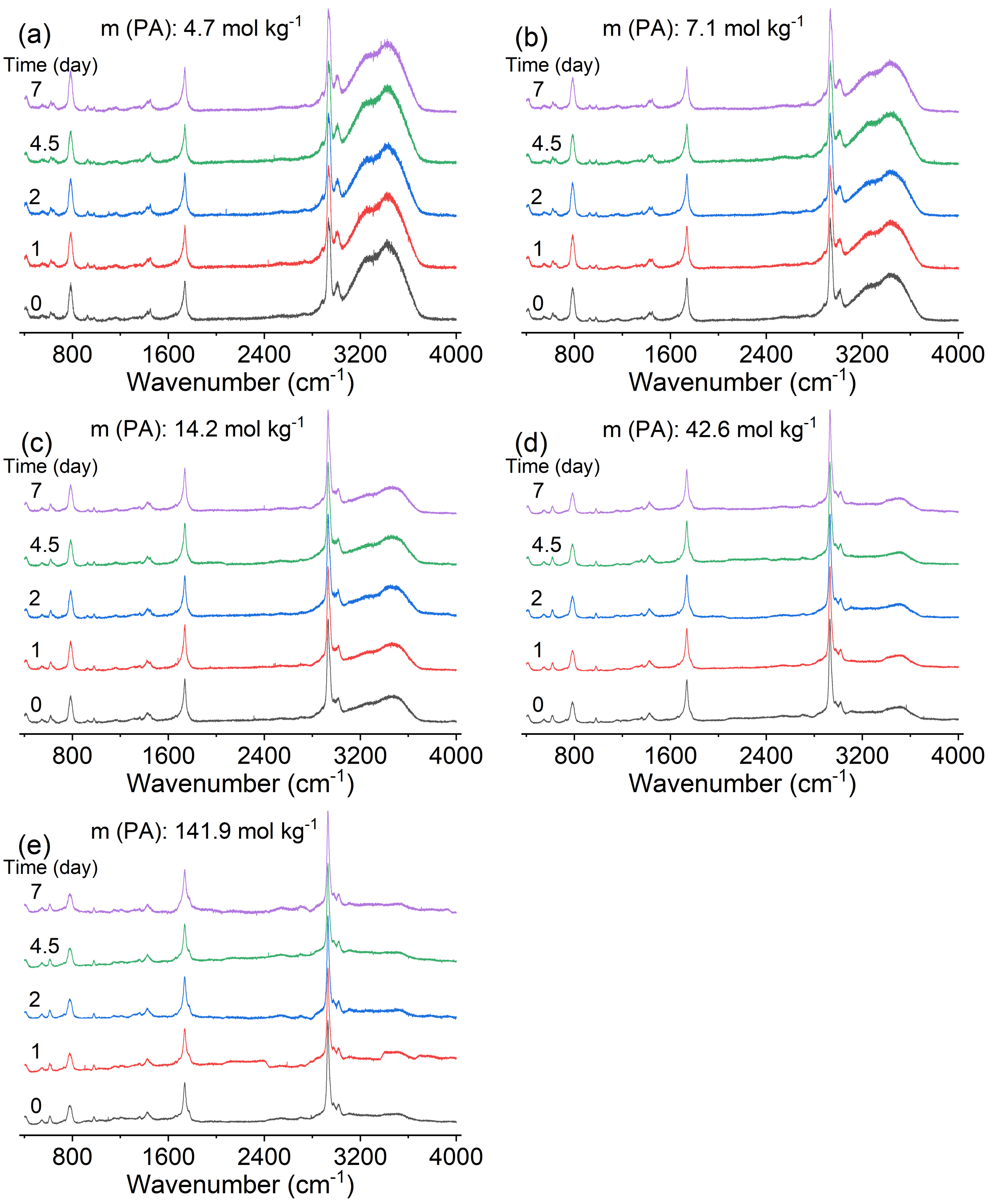

Figure S9. Raman spectra of PA bulk solutions with concentrations of (a) 4.7, (b) 7.1, (c) 14.2, (d) 42.6, and (e) 141.9 mol kg^{-1} in the dark from 0 to 7 days. As it has been reported that PA is surface active and m_{PA} at the air/water interface may be higher than that of the interior of the microdroplets.[8-10] To explore if the potential higher m_{PA} at the interface is the underlying reason for the surface reaction, we measured bulk solutions with m_{PA} ranging from 4.7 mol kg^{-1}, which is slightly higher than the droplet interior m_{PA} (mostly in the range of 4–4.6 mol kg^{-1} Figure 2a and 2b), to an extremely high value of 141.9 mol kg^{-1}. No significant bulk reactions were observed after 7 days even at these higher concentrations, suggesting that the potential enrichment of PA the at the interface alone cannot trigger the PA condensation reaction, and the reaction is likely initiated by the unique environment and potential the solute orientation/ structures at the air-water interface.

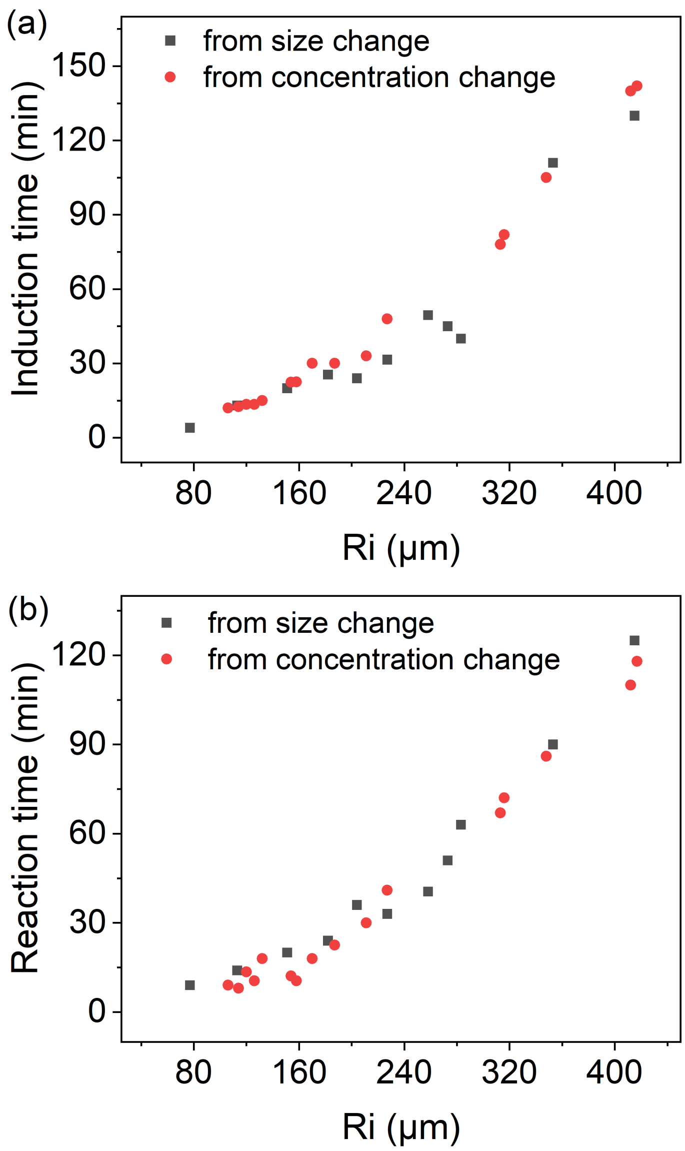

Figure S10. Comparison of the induction time (a) and reaction time (b) of PA reaction obtained from the evolution of droplet size (black) and the evolution of PA concentration (red).

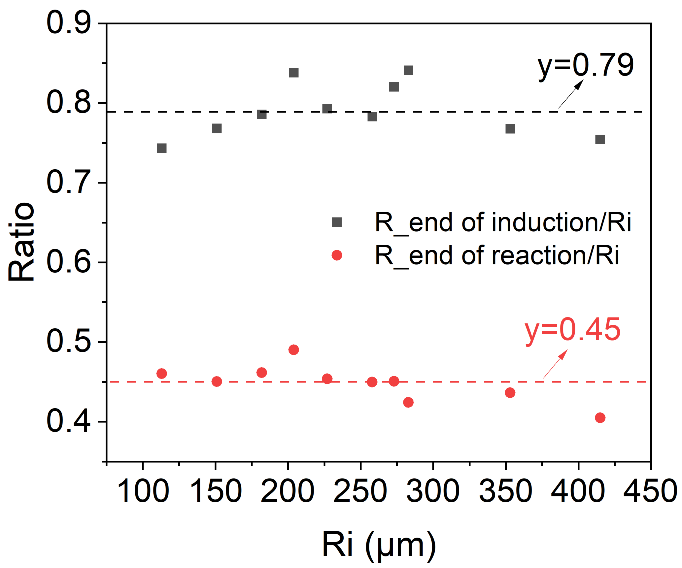

Figure S11. The ratio of the droplet size to the initial droplet size at the end of induction (R_end of induction/Ri) and at the end of reaction (R_end of reaction/Ri). R_end of induction/Ri has an average value of 0.79 with a standard deviation of 0.037, while R_end of reaction/Ri has an average value of 0.45 with a standard deviation of 0.023. The dashed lines, y=0.79 and y=0.45, indicate the average values of these two ratios.

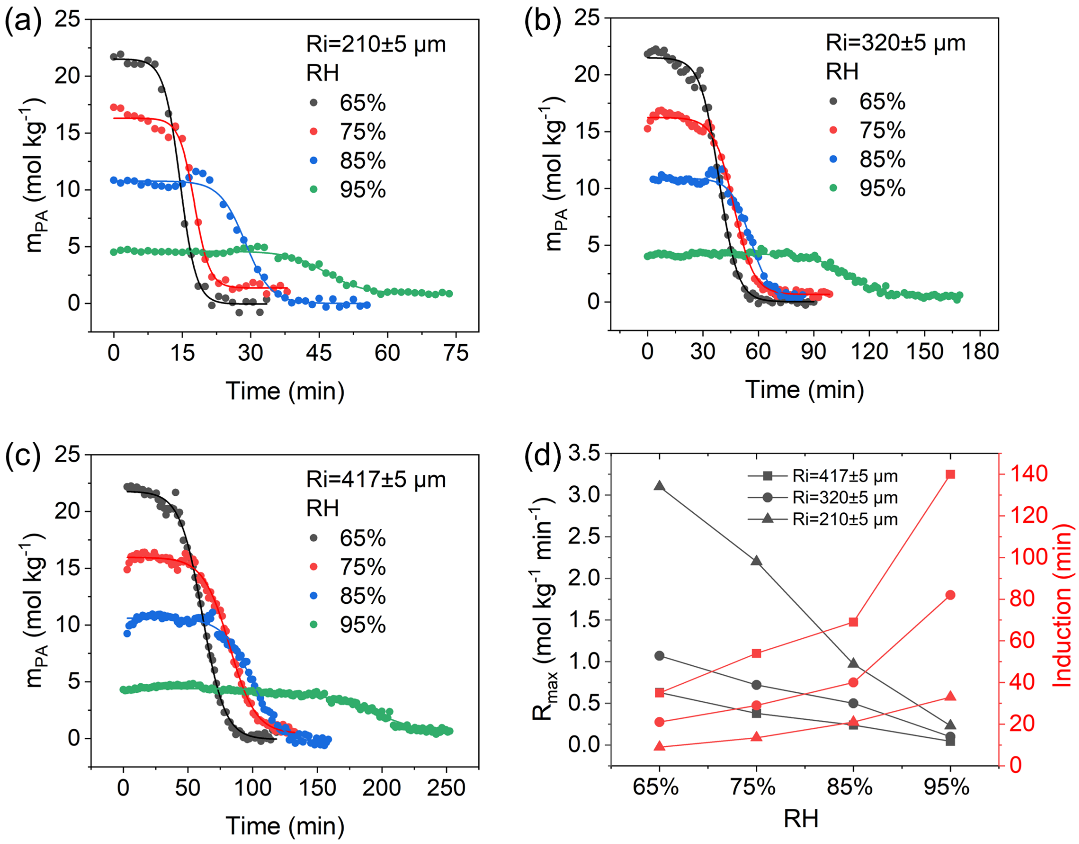

Figure S12. Time evolution of m_{PA} under different RH conditions: 65% (black), 75% (red), 85% (blue) and 95% (green) for droplets with Ri of (a) 210±5 μm, (b) 320±5 μm, and (c) 417±5 μm. (d) Changes of maximum apparent reaction rate (R_{max}) and induction time of droplets with different sizes as a function of RH. Figure S12a is the same as Figure 4 in the main text. It is worth noting that the accuracy of the high PA concentration (>15 mol kg^{-1}) determined for 65% and 75% RH might not be as precise as that less than 15 mol kg^{-1}. This is because the PA concentration calibration curve covers a range of 0.5 to 15 mol kg^{-1} (Figure S7) and the curve was extrapolated to determine the PA concentration greater than 15 mol kg^{-1}.

References

(1) Mael, L. E.; Busse, H.; Grassian, V. H. Measurements of immersion freezing and heterogeneous chemistry of atmospherically relevant single particles with micro-Raman spectroscopy. *Anal. Chem.* **2019**, *91* (17), 11138-11145.

(2) Heusser, K.; Heusser, R.; Jordan, J.; Urechie, V.; Diedrich, A.; Tank, J. Baroreflex curve fitting using a WYSIWYG Boltzmann sigmoidal equation. *Front. Neurosci.* **2021**, *15*.

(3) Petters, S. S.; Hilditch, T. G.; Tomaz, S.; Miles, R. E. H.; Reid, J. P.; Turpin, B. J. Volatility change during droplet evaporation of pyruvic acid. *ACS Earth Space Chem.* **2020**, *4* (5), 741-749.

(4) Perkins, R. J.; Shoemaker, R. K.; Carpenter, B. K.; Vaida, V. Chemical equilibria and kinetics in aqueous solutions of zymonic acid. *J. Phys. Chem. A* **2016**, *120* (51), 10096-10107.

(5) Deng, H.; Zheng, J.; Burgner, J.; Callender, R. Molecular properties of pyruvate bound to lactate dehydrogenase: a Raman spectroscopic study. *Proc. Natl. Acad. Sci. U.S.A.* **1989**, *86* (12), 4484-4488.

(6) Long, D.; George, W. Spectroscopic study of the pyruvate ion. *Trans. Faraday Soc.* **1960**, *56*, 1570-1581.

(7) Katon, J. E.; Covington, D. T. The vibrational spectra of crystalline sodium pyruvate. *Spectrosc. Lett.* **1979**, *12* (10), 761-766.

(8) Gordon, B. P.; Moore, F. G.; Scatena, L. F.; Richmond, G. L. On the rise: Experimental and computational vibrational sum frequency spectroscopy studies of pyruvic acid and its surface-active oligomer species at the air–water interface. *J. Phys. Chem. A* **2019**, *123* (49), 10609-10619.

(9) Kappes, K. J.; Deal, A. M.; Jespersen, M. F.; Blair, S. L.; Doussin, J.-F.; Cazaunau, M.; Pangui, E.; Hopper, B. N.; Johnson, M. S.; Vaida, V. Chemistry and photochemistry of pyruvic acid at the air–water interface. *J. Phys. Chem. A* **2021**, *125* (4), 1036-1049.

(10) Reed Harris, A. E.; Pajunoja, A.; Cazaunau, M.; Gratien, A.; Pangui, E.; Monod, A.; Griffith, E. C.; Virtanen, A.; Doussin, J.-F.; Vaida, V. Multiphase photochemistry of pyruvic acid under atmospheric conditions. *J. Phys. Chem. A* **2017**, *121* (18), 3327-3339.
